# Long COVID and Symptom Trajectory in a Representative Sample of Americans

**DOI:** 10.21203/rs.3.rs-1440503/v1

**Published:** 2022-03-16

**Authors:** Qiao Wu, Jennifer Ailshire, Eileen Crimmins

**Affiliations:** University of Southern California; University of Southern California; University of Southern California

## Abstract

People who have COVID-19 can experience symptoms for months. Studies on long COVID in the population lack representative samples and longitudinal data focusing on new-onset symptoms occurring with COVID while accounting for pre-infection symptoms. We use a sample representing the U.S. community population from the Understanding America Study COVID-19 Survey, which surveyed around 8,000 respondents bi-weekly from March 2020 to March 2021. Our final sample includes 308 infected individuals who were interviewed one month before, around the time of, and 12 weeks after infection. About 23% of the sample experienced new-onset symptoms during infection which lasted for more than 12 weeks, and thus can be considered as having long COVID. The most common persistent new-onset symptoms among those included in the study were headache (22%), runny or stuffy nose (19%), abdominal discomfort (18%), fatigue (17%), and diarrhea (13%). Long COVID was more likely among obese individuals (OR = 5.44, p < 0.001) and those who experienced hair loss (OR = 6.94, p < 0.05), headache (OR = 3.37, p < 0.05), and sore throat (OR = 3.56, p < 0.05) during infection. Risk was unrelated to age, gender, race/ethnicity, education, current smoking status, or comorbid chronic conditions. This work provides national estimates of long COVID in a representative sample after accounting for pre-infection symptoms.

## Introduction

While SARS-CoV-2 is usually thought of as an acute disease, we now know that some people with COVID experience a variety of post-acute health problems long after their disease onset. This experience of long-term persistent symptoms has been termed “long COVID” [1, 2]. Acute COVID typically lasts 3 weeks [2–4], but long COVID can last weeks, months, or longer [5]. Studies have reported widely varying prevalence levels of long COVID, ranging from 10% to over 90% (see Supplemental Table 1 for a summary of studies). Limitations in study design have made it difficult to obtain a clear estimate of the prevalence, symptoms of, and associated risk factors for long COVID in the United States population.

Early evidence of long COVID was based on discharged hospitalized COVID patients in a number of countries. Since these reflected patients with worse disease outcomes than average, the estimated prevalence of long COVID was generally high, ranging from 50–90% [6–11]. However, hospitalized patients account for a very small proportion (about 5%) of COVID-19 cases [12]; so, focusing only on samples of the discharged hospitalized patients provides a limited perspective on the experience of long COVID in the broader population. Studies using samples combining hospitalized and non-hospitalized individuals from specific geographic regions generally reported lower prevalence of long COVID compared to those focusing only on hospitalized patients, mostly ranging from 30–70% [1, 13–20]. Notably, the studies including a greater proportion of hospitalized patients, surveying more symptoms, and focusing on a shorter time frame generally report higher estimated long COVID prevalence. However, these estimates are not population representative.

Only two population representative surveys have been conducted to-date. In December 2020, the Office for National Statistics (ONS) estimated the prevalence of long COVID in the U.K. from a survey of 8,193 non-hospitalized and non-institutionalized respondents who ever tested positive for COVID during the survey follow-up − 21% exhibited symptoms lasting longer than 5 weeks, and 10% exhibited symptoms lasting longer than 12 weeks [21]. In June 2021, using data from the Real-time Assessment of Community Transmission-2 (REACT-2) Study, a nationally representative sample of the community population in England, Whitaker et al. [22] reported that among 28,713 respondents who reported a valid date of symptom onset, 38% experienced at least one symptom for more than 12 weeks. These two studies indicated lower prevalence of Long COVID prevalence compared to other previously discussed studies, and thus further suggest that study design has a large influence on the estimates. Importantly, these estimates are based on respondents’ symptoms reported after infection but did not consider symptoms prior to the infection.

Long COVID can affect multiple organs and body systems, and can be reflected in a wide range of long-lasting symptoms [4]. According to public health agencies [23, 24], the most commonly reported long COVID symptoms include respiratory abnormality, tiredness, neurocognitive problems, pain, flu-like symptoms, changes in smell or taste, as well as symptoms related to the cardiovascular system, digestive system, hair, and skin. Some symptoms of COVID are fairly common symptoms occurring also in non-COVID persons. Symptoms included in assessment of long COVID, such as fatigue, headache, body ache, sneezing, and dry skin, are common to other health conditions and may be linked to seasonal environmental conditions even among the healthy population. Thus, a potential limitation of previous studies is that they may have overestimated the prevalence of long COVID because they did not account for pre-infection symptoms. This limitation has been acknowledged in some earlier work [22, 25], but the lack of longitudinal data has thus far precluded a close examination of this.

In addition to interest in the population prevalence of long COVID, it is important to determine if long COVID risk differs across the population. Previous studies have found older adults [21, 25] and women [17, 22, 25, 26] to have elevated risks of long COVID. Existing health conditions may increase the risk of long COVID as well. The most commonly and consistently reported long COVID risk factor is obesity [22, 26, 27], which is also one of the strongest risk factors for severe COVID illness [28]. Pre-existing conditions in general [22, 25, 27] and asthma [27] are also found to be associated with higher risk of long COVID. Symptoms during acute COVID might predict long COVID as well. Augustin et al. [16] found the people experiencing anosmia and diarrhea are more likely to develop persistent symptoms. According to the CDC, in the U.S., socioeconomic and environmental factors are associated with increased risk of exposure to COVID, and these factors disproportionately affect racial and ethnic minority groups [29]. Though there is no direct evidence suggesting that those factors are associated with the risk of long COVID, racial/ethnic minorities and people with low education may be more at risk for long COVID as well.

In the current study, we used survey data from a nationally representative sample of community-dwelling U.S. adults, conducted from March 2020 to March 2021 to (1) estimate the baseline-symptom-adjusted prevalence of long COVID, (2) show the most commonly reported long COVID symptoms, and (3) identify the risk factors of becoming a COVID long hauler.

## Methods

### Data

We used data from the Understanding America Study (UAS) COVID-19 National Sample, an ongoing longitudinal national probability-based internet panel of approximately 9,000 non-institutional U.S. adults administered by the Center for Economic and Social Research (CESR), at the University of Southern California (USC). Respondents are recruited using a random selection of households from a postal service list of addresses, covering all households in the United States. They answer the survey using a computer, tablet, or smartphone; respondents were provided with a tablet and broadband internet if they did not have access to internet [30]. The UAS started administrating the longitudinal COVID-19 national survey to its panel members in March 2020 [31]. Follow-up surveys were fielded every two weeks beginning April 1, 2020. Over each 2-week survey period, one-fourteenth of the respondent pool was asked each day to fill out the survey within 2 weeks. More than 90% of the responses were completed in two weeks for each wave, and most were completed on the day of assignment. The UAS COVID national survey data are weighted to be nationally representative.

The current study used the first 25 waves of the survey, which were collected from March 10, 2020 to March 31, 2021. During this period, 8,425 respondents participated in the survey, and 872 people (~ 10% of the total participants) reported that they were diagnosed with, or tested positive for, COVID. We limited the analytic sample to 310 respondents who had COVID during the study period who also had information on self-reported symptoms at three times: 4 weeks before reporting a COVID diagnosis/positive test, at the time of the report of a COVID diagnosis/positive test, and 12 weeks after. After excluding 2 respondents with missing information on covariates, our final analytical sample consisted of 308 people. In other words, 564 were dropped due to missing data. We believe that the missingness is mostly random, since the characteristics are not significantly different at 95% confident level between our final sample and those we dropped (Supplemental Table 2). The only statistically significant difference is that those who had missing data are more likely to be Non-Hispanic others (p = 0.031). So, we were able to examine long COVID among a sample representing the majority of people who got COVID.

### Measures

#### COVID Diagnosis

The COVID diagnosed population was determined based on questions about both COVID tests and diagnosis. In the early period of the survey, tests were less available than in the later part of the period. Participants were asked: “Have you been tested for coronavirus since the last time you took our coronavirus survey? If so, what was the result?” and “Whether or not you have had a coronavirus test, has a doctor or another healthcare professional diagnosed you as having or probably having the coronavirus since the last time you took our coronavirus survey?”. We considered a respondent as having COVID who either tested positive to SARS-CoV-2 or who was diagnosed with COVID by a healthcare professional.

#### Self-Reported Symptoms

There is no official list of clinical symptoms defining long COVID. We examined the 18 self-reported symptoms included in the survey. At each wave, respondents were asked to report whether they had experienced the following symptoms in the past 7 days: (1) fever or chills, (2) runny or stuffy nose, (3) chest congestion, (4) cough, (5) sore throat, (6) sneezing, (7) muscle or body aches, (8) headaches, (9) fatigue or tiredness, (10) shortness of breath, (11) abdominal discomfort, (12) vomiting, (13) hair loss, (14) dry skin, (15) body temperature higher than 100.4 F or 38.0 C, (16) diarrhea, (17) lost sense of smell, (18) skin rash. The response options included “Yes”, “No”, and “Unsure”. We treated “Unsure” as not reporting the symptom. A symptom count variable ranging from 0 to 18 was then generated for each respondent at each wave by adding up the number of symptoms reported at a specific wave.

#### Long COVID

We define long COVID as symptoms reported by the respondent at the time of diagnosis, that were not present 4 weeks prior, and that lasted 12 weeks or more. If a respondent has any of the 18 symptoms at the time of diagnosis, did not experience the symptom 4 weeks before COVID, and reported the symptom 12 weeks after, the respondent is considered as having long COVID. This definition distinguishes symptoms most likely caused by COVID from symptoms the respondent was already experiencing prior to infection.

#### Existing Health Conditions

Because underlying medical conditions have been linked to elevated risk for severe illness from COVID-19 [32], we examine whether existing health conditions are associated with increased risk of long COVID. Participants were asked “Have you ever been told by a doctor, nurse, or other health professional that you have any of the following medical conditions?”: (1) diabetes, (2) cancer (other than skin cancer), (3) heart disease, (4) high blood pressure, (5) asthma, (6) chronic lung disease such as COPD or emphysema, (7) kidney disease, (8) autoimmune disorder such as rheumatoid arthritis or Crohn’s Disease, and (9) obesity. Each condition was treated as a binary variable in the analyses.

#### Other Covariates

Other covariates included age, gender, race/ethnicity, education level, and current smoking status. Age was categorized into three groups: ages 18 to 49, ages 50 to 64, and ages 65 and above. Race/ethnic groups included non-Hispanic White, non-Hispanic Black, Hispanic, and others. Education was classified as high school or less, some college education without a bachelor’s degree and a bachelor’s degree or more.

### Statistical Analysis

We treated the survey wave when the respondents reported that they tested positive for, or were diagnosed with, COVID as the time of infection. By design, the survey interval was 2 weeks. So, two waves (roughly 4 weeks) prior to the infection were the pre-infection time, and 6 waves (roughly 12–13 weeks) after infection was the post-infection stage.

We first summarized the sample characteristics at the time of reported infection and compared them to the profile of the COVID-infected population in the United States provided by the Centers for Disease Control [33] in order to assess the generalizability of the survey results. We also compared the characteristics of the long COVID group to those who experienced COVID but not long COVID.

Next, we estimated the prevalence of long COVID using both the reported current symptoms, to compare with estimates from previous studies, as well as new symptom onset (accounting for pre-infection symptoms). We compared our estimates to other nationally representative studies.

Then, we compared the proportions of our analytical sample who reported each of the symptoms at pre-infection, infection, and post-infection stages to show the overall recovery of the infected population. And after that we further compared the proportions of those with long COVID in our sample who reported each of the symptoms at each stage to show how their symptoms changed over time, and the frequency of reported symptoms. Based on our long COVID definition, we also showed the ranking of persistent new-onset symptoms among those with long COVID.

Lastly, logistic regression models were used to identify sociodemographic and health-related risk factors associated with long COVID: smoking, existing health conditions, and new-onset symptoms at the time of infection.

All results were weighted to be nationally representative, adjusting for differential sampling probabilities and survey nonresponse. All analyses were performed using STATA Version 16.0. Informed consent was obtained during the response from participants. The current analysis used the STROBE (STrengthening the Reporting of OBservational studies in Epidemiology) cohort reporting guidelines.

## Results

### Sample Characteristics at the Time of Infection

Table 1 shows sample characteristics at the time of reported SARS-CoV-2 infection. Our final sample had a mean age of 46 (third column of Table 1); More than half of the sample was female (57%); 61% was non-Hispanic White; 12% were non-Hispanic Black; and 22% were Hispanic. Both our final sample and the UAS sample of all COVID cases (the second column of Table 1) are very similar in age, gender, and racial/ethnic distribution to COVID cases tracked by the CDC during the same timeframe? [33] (the first column of Table 1).

In the final sample, about 41% had high school or less than high school completion, 35% reported some college, and 24% had a bachelor’s degree or higher. Almost 30% of the respondents were current smokers. In terms of existing health conditions, 18% had diabetes, 5% had cancer, 9% had heart disease, 29% had high blood pressure, 19% had asthma, 5% had chronic lung disease, 4% had kidney disease, 5% had an autoimmune disorder, and 24% were obese. Half of the sample had none of the underlying conditions.

More than two fifths of the sample reported new-onset body aches (45%), fatigue (43%), cough (41%), and headache (40%) at the infection stage; in addition, more than one fourth had new-onset fever (37%), runny or stuffy nose (35%), loss of smell (33%), diarrhea (29%), sore throat (28%), shortness of breath (26%), and chest congestion (25%).

At the time of infection, 80% of the respondents were symptomatic, and the average symptom count was 6. Both the proportion symptomatic and the average symptom count were fairly similar between the UAS total COVID sample (the second column of Table 1) and our final analytical sample (the third column of Table 1). Persons with long COVID had more symptoms on average than those who recovered quickly (7.9 vs 5.4).

Compared to people who did not experience long COVID, the long haulers were significantly more likely to be obese (p = 0.004). In terms of new-onset symptoms, the long haulers were more likely to experience headache (0.004), fever (0.037), and runny or stuffy nose (0.034).

### The Prevalence of long COVID

In our sample of 308 COVID-infected respondents, 40% experienced at least one symptom twelve weeks later, and this would have been the estimated prevalence of long COVID if pre-infection symptoms were not considered. However, after accounting for pre-infection symptoms, only 23% of the infected experienced at least one new-onset COVID symptom that lasted for at least 12 weeks. These estimates can be compared to the other nationally representative prevalence of long COVID estimated for UK by ONS and England by Whitaker et al. of 10% and 38% respectively. Our long COVID prevalence (40%) among the U.S. population without accounting for pre-infection symptom level, is very similar to that made by Whitaker et al. of 38%. and after controlling for pre-infection symptoms, our estimate is between the other two.

### Symptoms Trend and Most Reported Symptoms

**Figure 1:**
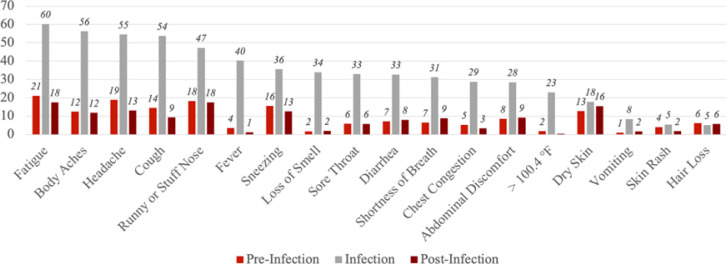
Percent with Self-Reported Symptoms at Pre-Infection, Infection, and Post-Infection Stages, among the COVID-Infected (n=308) Notes The pre-infection stage is 4 weeks before the COVID diagnosis or positive test. The infection stage is the time of COVID diagnosis or positive test. The post-infection stage is 12 weeks after the COVID diagnosis or positive test. Symptoms were listed based on the proportion reported at the time of infection.

Figure 1 shows the proportions of our sample reporting each of the symptoms at pre-infection, infection, and post-infection stages. Among the infected, more than half experienced fatigue (60%), body aches (56%), headache (55%), and cough (54%) at the time of infection. For most of the symptoms, the proportions are elevated at the time of infection, but overall, tended to return to pre-infection levels at the post-infection stage.

**Figure 2:**
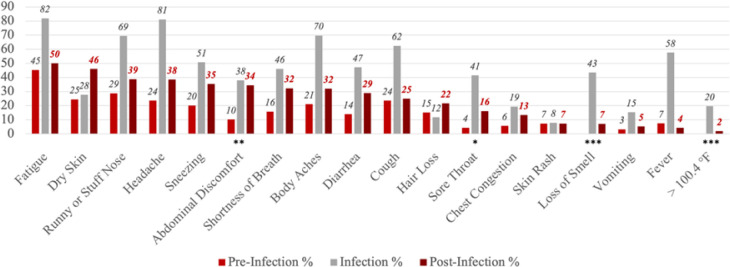
Percent with Self-Reported Symptoms at Pre-Infection, Infection, and Post-Infection Stages among COVID Long haulers (n=74) Notes The pre-infection stage is 4 weeks before the COVID diagnosis or positive test. The infection stage is the time of COVID diagnosis or positive test. The post-infection stage is 12 weeks after the COVID diagnosis or positive test. Symptoms were listed based on the proportion reported at the post-infection stage. The statistical significance of the difference in proportions between pre-infection stage and post-infection stage is indicated for Panel B. * p<0.05 ** p<0.01 *** p<0.001

Similarly, Fig. 2 shows the proportions reporting each of the symptoms at the three stages, but only among the COVID long haulers (n = 74). For many symptoms, the proportions peaked at the time of infection and then dropped but remained higher at the post-infection stage compared to pre-infection stage. Specifically, relative to the pre-infection level, the proportion reporting abdominal discomfort (p = 0.004), sore throat (p = 0.048), loss of smell (p < 0.001), and having a body temperature higher than 100.4°F (p < 0.001) were statistically significantly higher at the post-infection stage. Among those with long COVID, the most commonly reported symptoms at the post-infection stage included fatigue (50%), dry skin (46%), runny or stuffy nose (39%), headache (38%), and sneezing (35%). It is important to note that these most reported symptoms did not account for pre-infection baseline level, and it is possible that they were commonly reported partially because they had high prevalence even without SARS-CoV-2 infection.

**Figure 3:**
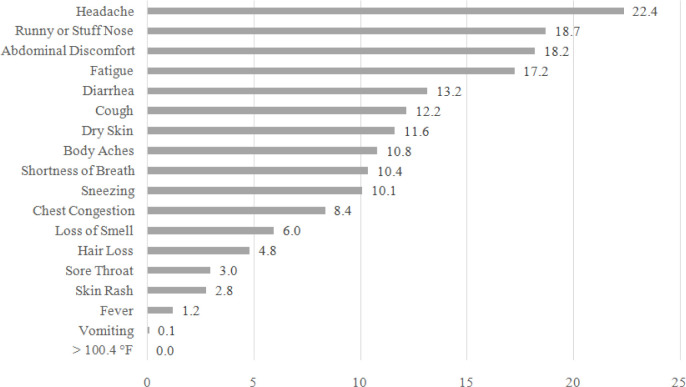
Persistent New-Onset COVID Symptoms among those with Long COVID 12 Weeks after Infection

To account for the pre-infection prevalence of the symptoms, Fig. 3 shows the prevalence of only persistent new-onset symptoms among those with long COVID at the post-infection stage. Because the long haulers started experiencing these symptoms at the time of infection, they are more likely to be related to COVID specifically. The most reported persistent new-onset symptoms were headache (22%), runny or stuffy nose (19%), abdominal discomfort (18%), fatigue (17%), and diarrhea (13%). In terms of both the ranking and the prevalence, many symptoms in Fig. 3 are different from Fig. 2. For example, the rankings of dry skin (No.2 to No.7) and sneezing (No.5 to No.10) dropped markedly, while the rankings of diarrhea (No.9 to No.5) and cough (No.10 to No.6) notably increased.

### Predictors of Long COVID

Table 2 shows the logistic regression model predicting long COVID among our sample of 308 SARS-CoV-2 infected respondents. People who were obese (OR = 5.44, p < 0.001), and who experienced hair loss (OR = 6.94, p = 0.047), headache (OR = 3.37, p = 0.023), and sore throat (OR = 3.56, p = 0.021) at the time of infection, had significantly higher odds of experiencing long COVID. On the contrary, the odds among people who experienced chest congestion (OR = 0.09, p < 0.001) were lower. None of the existing chronic health conditions were related to having long COVID. The odds were not significantly different across demographic and education groups in either the full model or the model unadjusted for other covariates.

## Discussion

### Main Findings

Our results indicate that the estimated prevalence of long COVID in a population representative sample differs depending on whether pre-infection symptoms are accounted for. In the U.S. population, most people with COVID return to their pre-infection symptom level after the acute phase of the disease. However, more than one-fifth (23%) experience long COVID, with at least one symptom originating around the time of SARS-CoV-2 infection lasting for more than 12 weeks. Without adjusting for pre-infection symptoms, the prevalence is estimated to be 40%, which suggests the potential for a significant over-estimation of long COVID in previous studies.

The most frequently experienced persistent new-onset symptoms among those with long COVID include headache (22%), runny or stuffy nose (19%), abdominal discomfort (18%), fatigue (17%), and diarrhea (13%). The fully adjusted logistic regression model indicates that the likelihood of experiencing long COVID is not significantly associated with sociodemographic or behavioral factors including age, gender, race/ethnicity, education, current smoking status or the presence of chronic conditions. COVID long haulers are more likely to experience hair loss, headache, and sore throat at the time of infection compared to their counterparts whose symptoms reduce more quickly. Also, those who are obese are at higher risk of experiencing persistent new-onset symptoms.

To our knowledge, this is the first study that defined long COVID accounting for pre-infection baseline symptoms. Even before SARS-CoV-2 infection, more than two-fifths (44%) of our sample experienced at least one symptom that can be potentially linked to COVID. Also, among the infected, the prevalence of most symptoms returned to the pre-infection level at the post-infection stage. It means that people report many symptoms both before and after COVID that may be due to other conditions. So, while around 40% of the COVID-infected persons have at least one symptom 12 weeks after COVID diagnosis, this may overestimate the prevalence of long COVID if these persons are all classified as long COVID. The longitudinal nature of data, from pre-infection to post-infection stage, made it possible to distinguish new onset symptoms from the symptoms that might be experienced by someone without SARS-CoV-2 infection. Admittedly, the current approach only picks up on new-onset symptoms and not the changing severity of symptoms. But still, due to the relatively high prevalence of symptoms in our sample even before COVID, our longitudinal and conservative approach can help avoid possible overestimation.

Compared to the estimates of long COVID prevalence based on other nationally representative studies, our final estimate (23%) based on UAS data is between the U.K. ONS estimate (10%) [21], and the Whitaker et al. estimate (38%) [22]. The three studies are similar in study design and population representativeness, so the difference in estimates may reflect to the different number of symptoms used in each study. Specifically, the ONS estimate is based on 12 symptoms, while the Whitaker et al. estimate on 29 symptoms. The current study included 18 symptoms, which is roughly between the other two studies. The symptoms included in REACT-2 but not in UAS include sudden swelling to face or lips, sore eyes, purple scores/blisters on feet, numbness/tingling, hoarse voice, heavy arms/legs, dizziness, difficulty sleeping, chills, and appetite loss. However, these symptoms generally have low prevalence among the SARS-CoV-2 infected, and/or diminish quickly after initial infection [22]. Hence, the lack of these symptoms in the questionnaire is not likely to cause a significant difference in the estimated prevalence of long COVID.

Our estimated prevalence is also similar to the estimate of 27% based on never-hospitalized COVID symptomatic Californians [17], and the estimate of 30% based on a sample combining hospitalized patients and outpatients in Seattle, Washington [1]. While it is notably lower than the estimated prevalence of at least 50% using hospitalized patient sample in Michigan [8]. These differences may reflect the fact that we adjust for pre-infection symptoms, and we do not represent the hospitalized population.

The significant association between long COVID and obesity is consistent with previous studies [22, 25, 26]. Both Whitaker et al.’s and the ONS studies found that existing health conditions are associated with elevated long COVID risk. Our results do not show any link between the presence of health conditions and long COVID.

We differ from some existing studies, in that we did not find a significant association between long COVID and any sociodemographic factors included in this study. It is probably because the analytic approaches used by the ONS [21], Whitaker et al. [22], and Sudre et al. [26] to assess risk factors for long COVID either are based on bivariate comparisons, or do not include the effects of existing health conditions as we do. Hence, the age differences and gender differences they found may be explained by health differences across gender and age groups or other uncontrolled factors. Also, Sudre et al. collected data from an international sample including respondents from the U.K., the U.S., and Sweden, while Whitaker et al. focus on England and the ONS focused on the U.K. population. The discrepancy in results may also reflect differences in socioeconomic and demographic context across countries. We found some symptoms reported at the time of infection to be associated with experiencing long COVID, but the symptoms we found (hair loss, headache, and sore throat) are different from the ones identified by Augustin et al. [16] (anosmia and diarrhea). It is probably because we used new-onset symptoms as the predictors in our regression model, but the previous study was not able to distinguish new-onset symptoms from those started even before SARS-CoV-2 infection.

By limiting our sample to those who had complete data at the pre-infection stage, the time of infection, and post-infection stage, we excluded those who were diagnosed in the last 5 waves of the survey. It means that for the respondents in our sample, the latest possible date of diagnosis was between November 25 to December 23, 2020 (the 19th survey wave). Since COVID vaccines were available for only a small number of health care workers at that time, we do not think the vaccination would change our estimate in any meaningful way, so we did not include information on vaccination status.

### Limitations

Our study has limitations. We are likely to have missed some severe COVID cases since they likely would not have answered the survey while suffering from severe illness. Information on hospitalization is not available in the UAS. Since hospitalized COVID patients generally experience moderate or severe disease outcomes, it is reasonable to assume that they are more likely to have missing data in the UAS and to be excluded from our final sample. This would lead to an underestimate of the prevalence of long COVID. Given that around 5% of the SARS-CoV-2 infected population are hospitalized [12], and since long COVID is highly prevalent (50%−90%) among hospitalized patients [6–11], we believe that the real prevalence at population level may be higher than our estimate of 23%, ranging from 24–26%. Some limitations of our study are due to the nature of the secondary data we use. The UAS COVID National Survey does not have information on brain fog, which is considered to be a long COVID symptom. So, we failed to include the COVID long haulers who suffered from only persistent brain fog. Finally, Our assessment of long COVID is based on self-reports, instead of clinical diagnoses, and we do not have a clear set of clinical indicators of long COVID. However, self-reported symptoms are still valuable for gaining insights into what is happening in the population. but it provides little information on the pathology or mechanism.

With the availability of vaccines and the onset of new variants, the nation has moved into new stages of the pandemic. The vaccinated population has tripled since the last wave of data used in the current study, and by March 2022, more than 65% of the total US population have been fully vaccinated [34]. The Omicron variant, a variant which spreads more easily than the original virus and the Delta variant [35], emerged in the US in December 2021 and by February 2022, almost all the new cases were driven by Omicron lineages [36]. It remains unclear how vaccination affects long COVID [4], and there is limited evidence on whether the Omicron wave has changed what we know about long COVID [37, 38]. Nevertheless, long COVID is still a public health concern. More knowledge on its prevalence, persistent symptoms, and risk factors may help healthcare professionals allocate resources and services to help long haulers get back to normal lives.

## Supplementary Material

Supplement 1

## Figures and Tables

**Table 1:** Baseline Sample Characteristics

	CDC COVID Cases	COVID Population in UAS	Final Sample	People with Long COVID	People without Long COVID	P Values
	n = 21,926,390	n = 872	n = 308	n = 74	n = 234	n = 308
Covariates	% / *mean*	% / *mean*	% / *mean*	% / *mean*	% / *mean*	With VS Without Long COVID
**Age (mean)**	-	*45.9*	*46.0*	*44.9*	*46.4*	0.555
18–49	60.7	60.2	56.5	56.8	56.5	0.973
50–64	23.2	27.4	29.9	34.4	28.6	0.498
65+	16.1	12.4	13.6	8.9	14.9	0.209
**Gender**						
Male	47.8	44.0	42.7	34.9	45.0	0.270
Female	52.2	56.0	57.3	65.1	55.0	0.270
**Race/Ethnicity**						
Non-Hispanic White	55.9	55.7	60.6	62.3	60.1	0.819
Non-Hispanic Black	12.2	11.5	12.2	4.1	14.5	0.190
Hispanic	20.7	24.0	22.4	30.0	20.1	0.271
Non-Hispanic Others	11.3	8.8	4.9	3.6	5.3	0.586
**Education**						
High School and Less	-	39.4	40.9	32.3	43.4	0.253
Some College	-	32.6	35.1	39.0	34.0	0.553
College and More	-	28.0	24.0	28.7	22.6	0.424
**Current Smoker**	-	24.8	29.4	19.7	32.2	0.106
**Health Conditions**						
Diabetes	-	13.6	17.7	20.0	17.1	0.694
Cancer	-	4.8	5.2	1.1	6.4	0.059
Heart Disease	-	6.7	9.2	7.2	9.8	0.540
Hypertension	-	30.7	28.6	33.3	27.2	0.468
Asthma	-	15.3	18.9	24.1	17.3	0.372
Chronic Lung Disease	-	4.2	4.6	6.8	4.0	0.850
Kidney Disease	-	3.5	4.4	7.5	3.5	0.363
Autoimmune Disorder	-	5.1	4.7	9.2	3.4	0.055
Obesity	-	20.1	24.2	**42.4**	**18.9**	**0.004** **
**New-Onset Symptoms at Infection Stage**						
Body aches	-	-	44.5	50.3	42.8	0.413
Fatigue	-	-	43.0	47.1	41.7	0.554
Cough	-	-	40.8	42.7	40.3	0.791
Headache	-	-	40.4	**60.0**	**34.7**	**0.004** **
Fever	-	-	37.3	**51.7**	**33.1**	0.037*
Runny or stuffy nose	-	-	34.8	**49.3**	**30.6**	0.034*
Lost of smell	-	-	32.7	43.5	29.6	0.110
Diarrhea	-	-	28.5	37.7	25.9	0.168
Sore throat	-	-	28.0	40.1	24.5	0.061
Shortness of breath	-	-	26.0	34.9	23.4	0.177
Chest congestion	-	-	25.2	17.2	27.6	0.116
Sneezing	-	-	24.1	33.8	21.2	0.104
> 100.4°F	-	-	22.7	19.6	23.6	0.616
Abdominal discomfort	-	-	22.3	30.1	20.0	0.187
Dry skin	-	-	11.2	15.6	10.0	0.327
Vomiting	-	-	7.8	13.7	6.1	0.167
Skin rash	-	-	4.0	3.7	4.1	0.903
Hair loss	-	-	2.6	6.3	1.5	0.100
**Symptomatic When Diagnosed**	-	83.2	80.3	100.0	74.6	-
**Symptom Count When Diagnosed (mean)**	-	*5.8*	*6.0*	*7.9*	*5.4*	0.000***

Note.

p < 0.05

** p < 0.01

*** p < 0.001

The CDC COVID cases information were from CDC COVID Data Tracker: https://covid.cdc.gov/covid-data-tracker/#demographics

For the UAS sample, the time of COVID diagnosis is considered baseline, and all percentages and means are weighted to be nationally representative.

**Table 2:** Logistic Regression Model Predicting Long COVID

n = 308	Odds Ratio		P	95% CI
**Age Categories - Ref: 18–44**				
45–64	0.76		0.604	[0.272,2.131]
65+	0.94		0.922	[0.261,3.375]
**Male**	0.93		0.849	[0.456,1.907]
**Race/Ethnicity - Ref: Non-Hispanic White**				
Non-Hispanic Black	0.46		0.420	[0.068,3.069]
Hispanic	0.72		0.464	[0.303,1.724]
Non-Hispanic Others	0.50		0.469	[0.077,3.250]
**Education Categories - Ref: High School and Less**				
Some College	1.45		0.412	[0.595,3.553]
College and More	1.38		0.531	[0.503,3.789]
**Current Smoker**	0.74		0.541	[0.283,1.940]
**Existing Conditions**				
Obesity	5.44	***	< 0.001	[2.120,13.960]
Diabetes	1.03		0.967	[0.303,3.475]
Cancer	0.10		0.065	[0.008,1.158]
Heart Disease	0.21		0.119	[0.031,1.482]
High Blood Pressure	1.38		0.492	[0.553,3.431]
Asthma	0.96		0.951	[0.255,3.617]
Chronic Lung Disease	3.05		0.443	[0.177,52.768]
Kidney Disease	1.28		0.797	[0.190,8.679]
Autoimmune Disorder	1.83		0.354	[0.508,6.618]
**New-Onset Symptoms at Infection Stage**				
Abdominal discomfort	1.26		0.591	[0.542,2.931]
Body aches	1.25		0.688	[0.418,3.743]
Chest congestion	0.09	***	< 0.001	[0.023,0.345]
Cough	0.54		0.243	[0.191,1.521]
Diarrhea	1.02		0.958	[0.459,2.274]
Dry skin	1.10		0.890	[0.289,4.182]
Fatigue	0.46		0.184	[0.146,1.446]
Fever	1.06		0.901	[0.400,2.831]
Hair loss	6.94	*	0.047	[1.028,46.916]
Headache	3.37	*	0.023	[1.181,9.604]
Lost of smell	1.58		0.256	[0.717,3.496]
Runny or stuffy nose	1.38		0.540	[0.494,3.839]
Skin rash	0.53		0.427	[0.109,2.552]
Shortness of breath	1.70		0.380	[0.519,5.576]
Sneezing	1.56		0.380	[0.576,4.242]
Sore throat	3.56	*	0.021	[1.212,10.460]
Vomiting	0.75		0.658	[0.216,2.635]

Notes

Coefficients are reported in odds ratios

* p < 0.05

** p < 0.01

*** p < 0.001

95% confidence intervals are reported in brackets

## Data Availability

The survey and data are available from University of Southern California Understanding America Study website: https://uasdata.usc.edu/index.php
