# Supplementary material for: Long COVID and Symptom Trajectory in a Representative Sample of Americans
Source: Res Sq. 2022 Mar 16:rs.3.rs-1440503. Preprint. [Version 1] doi: 10.21203/rs.3.rs-1440503/v1 (PMC8936101; doi:10.21203/rs.3.rs-1440503/v1)
Supplement: Supplement 1 [file 550374cdac5b8879c4f2d9b3.docx]

**Supplementary Information**

**Supplementary Table 1. Previous Estimates of the Prevalence of Long COVID**

| Sample Type | Place | Study | Sample Info | Follow-Up Time | Prevalence of Long COVID |
| --- | --- | --- | --- | --- | --- |
| Discharged Hospitalized Patients Only | Italy | Carfi et al. 2020 | N=143; Patient sample from one hospital; All hospitalized. | 8 weeks (mean) | 87% |
|  | France | Garrigues et al. 2020 | N=120; Patient sample from one hospital; All hospitalized. | 16 weeks (mean) | At least 55% |
|  | US | Chopra et al. 2020 | N=488; Patient sample from 38 hospitals in Michigan; All hospitalized. | 8 weeks | At least 50% |
|  | UK | Halpin et al. 2020 | N=100; Patient sample form one hospital; All hospitalized. | 7 weeks (mean) | At least 60% |
|  | China | Huang C et al. 2021 | N=1,733; Patient sample from one hospital; All hospitalized. | 27 weeks (mean) | 76% |
|  | China | Zhao et al. 2020 | N=55; Patient sample from 3 hospitals; All hospitalized. | 12 weeks | 71% |
| Both Discharged Hospitalized Patients and Non-Hospitalized COVID Individuals | International | Davis et al. 2021 | N=3,762; Internet sample from 56 countries, 35% outpatients, 8% hospitalized. | 35 weeks | 91% |
|  | International | Goërtz et al. 2020 | N=2,113; Facebook Long COVID Support Group Members from Netherlands and Belgium; 5% hospitalized. | 12 weeks | At least 87% |
|  | International | Taquet et al. 2021 | N=273,618; Medical records of patients who are diagnosed to have COVID mainly from US and also from India, Australia, Malaysia, Taiwan, Spain, UK, and Bulgaria. About 20% hospitalized. | 12-24 weeks | 37% |
|  | International | Sudre et al. 2021 | N=4,182; Internet sample from UK, US, and Sweden; 14% Had Hospital COVID Visit. | 12 weeks | 2% |
|  | France | Carvalho-Schneider et al. 2020 | N=130; Patient sample from one hospital; 65% outpatients; **35% hospitalized**. | 8 weeks | 66% |
|  | Germany | Seeßle et al. 2021 | N=96; Patient sample from one hospital, 68% outpatients, **32% hospitalized**. | 48 weeks | 77% |
|  | Norway | Blomberg et al. 2021 | N=312; People Positively Tested in the City of Bergen or Patients Admitted to 2 City's Hospitals; 79% Home-Isolated; **21% Hospitalized**. | 24 weeks | 61% |
|  | US | Logue et al. 2021 | N=177; Positively Tested People in Seattle; 6% Asymptomatic; 85% Outpatients; **9% Hospitalized**. | Up to 36 weeks | 30% |
|  | Germany | Augustin et al. 2021 | N=958; Positively Tested Outpatients at Univeristy Hospital Cologne in Germany; **All Never-Hospitalized.** | 28 weeks | 35% |
|  | US | Huang Y et al. 2021 | N=1,407; COVID Patients Have Electronic Records within the University of California System; **All Never-Hospitalized**. | 8 weeks | 27% |
|  | US | Tenforde et al. 2020 | N=274; Outpatients from 14 US Academic Health Care Systems in 13 States; **All Never-Hospitalized**. | 2 weeks (median) | At least 50% |
|  | US | Bell et al. 2021 | N=303; Positively tested people from Arizona; **All Never-Hospitalizaed** | 8 weeks | 77% |
|  | Israel | Klein et al. 2021 | N=103; Mild COVID patients in Israel recruited via social media; **All Never Receiving Respiratory Support or Intensive Care Unit Admission.** | 24 weeks | 46% |
| Population Representative | UK | ONS 2020 | - | 12 weeks | 10% |
|  | England | Whitaker et al. 2021 | N=76,155; Random Population Sample of Individuals of Symptomatic Acute COVID; 63% Never Sought Medical Attention; 37% Sought Medical Attention Outside Hospital; **Less than 1% Admitted to Hospital**. | 12 weeks | 38% |

Notes

The follow-up time has been converted to weeks for easier comparison.

COVID Outpatients are not considered as hospitalized patients.

For all previous studies, the prevalence of long COVID is estimated by the proportion of still symptomatic individuals, except for the study done by Zhao et al. 2020, which estimates it using the proportion of those having radiological abnormalities.

**Supplementary Table 2. Difference between Final Sample and Dropped Participants**

|  | **COVID Population in UAS** | **Final Sample** | **Dropped** | **T-Test P Values** |
| --- | --- | --- | --- | --- |
|  | n = 872 | n = 308 | n = 564 | n = 308 |
| **Covariates** | % / *mean* | % / *mean* | % / *mean* | Final Sample VS Dropped |
| **Age (mean)** | *45.9* | *46.0* | *45.8* | 0.897 |
| 18-49 | 60.2 | 56.5 | 62.1 | 0.229 |
| 50-64 | 27.4 | 29.9 | 26.1 | 0.366 |
| 65+ | 12.4 | 13.6 | 11.8 | 0.537 |
| **Gender** |  |  |  |  |
| Male | 44.0 | 42.7 | 44.7 | 0.674 |
| Female | 56.0 | 57.3 | 55.3 | 0.674 |
| **Race/Ethnicity** |  |  |  |  |
| Non-Hispanic White | 55.7 | 60.6 | 53.1 | 0.136 |
| Non-Hispanic Black | 11.5 | 12.2 | 11.1 | 0.757 |
| Hispanic | 24.0 | 22.4 | 24.9 | 0.588 |
| Non-Hispanic Others | 8.8 | 4.9 | 10.9 | **0.031*** |
| **Education** |  |  |  |  |
| High School and Less | 39.4 | 40.9 | 38.7 | 0.647 |
| Some College | 32.6 | 35.1 | 31.3 | 0.378 |
| College and More | 28.0 | 24.0 | 30.1 | 0.138 |
| **Current Smoker** | 24.8 | 29.4 | 22.4 | 0.105 |
| **Health Conditions** |  |  |  |  |
| Diabetes | 13.6 | 17.7 | 11.4 | 0.059 |
| Cancer | 4.8 | 5.2 | 4.5 | 0.745 |
| Heart Disease | 6.7 | 9.2 | 5.4 | 0.116 |
| Hypertension | 30.7 | 28.6 | 31.9 | 0.451 |
| Asthma | 15.3 | 18.9 | 13.3 | 0.124 |
| Chronic Lung Disease | 4.2 | 4.6 | 4.0 | 0.757 |
| Kidney Disease | 3.5 | 4.4 | 3.0 | 0.468 |
| Autoimmune Disorder | 5.1 | 4.7 | 5.4 | 0.701 |
| Obesity | 20.1 | 24.2 | 17.8 | 0.094 |
| **Symptomatic When Diagnosed** | 83.2 | 80.3 | 84.7 | 0.257 |
| **Symptom Count When Diagnosed (mean)** | *5.8* | *6.0* | *5.8* | 0.698 |

Notes

* p<0.05 ** p<0.01 *** p<0.001
